# Abrogation of Marek’s disease virus replication using CRISPR/Cas9

**DOI:** 10.1038/s41598-020-67951-1

**Published:** 2020-07-02

**Authors:** Ibrahim T. Hagag, Darren J. Wight, Denise Bartsch, Hicham Sid, Ingo Jordan, Luca D. Bertzbach, Benjamin Schusser, Benedikt B. Kaufer

**Affiliations:** 1 Institut für Virologie, Freie Universität Berlin, Robert-von-Ostertag-Str. 7-13, 14163 Berlin, Germany; 2 Department of Virology, Faculty of Veterinary Medicine, Zagazig University, El-Tagneed St. 114, Zagazig, 44511 Egypt; 3 Reproductive Biotechnology, School of Life Sciences Weihenstephan, Technical University of Munich, Liesel-Beckmann-Str. 1, 85354 Freising, Germany; 4 ProBioGen AG, Herbert-Bayer-Straße 8, 13086 Berlin, Germany

**Keywords:** Virology, Tumour virus infections, Pathogens

## Abstract

Marek’s disease virus (MDV) is a highly cell-associated alphaherpesvirus that causes deadly lymphomas in chickens. While vaccination protects against clinical symptoms, MDV field strains can still circulate in vaccinated flocks and continuously evolve towards greater virulence. MDV vaccines do not provide sterilizing immunity, allowing the virus to overcome vaccine protection, and has increased the need for more potent vaccines or alternative interventions. In this study, we addressed if the CRISPR/Cas9 system can protect cells from MDV replication. We first screened a number of guide RNAs (gRNAs) targeting essential MDV genes for their ability to prevent virus replication. Single gRNAs significantly inhibited virus replication, but could result in the emergence of escape mutants. Strikingly, combining two or more gRNAs completely abrogated virus replication and no escape mutants were observed upon serial passaging. Our study provides the first proof-of-concept, demonstrating that the CRISPR/Cas9 system can be efficiently used to block MDV replication. The presented findings lay the foundation for future research to completely protect chickens from this deadly pathogen.

## Introduction

Marek’s disease virus (MDV) is a cell-associated alphaherpesvirus that infects chickens and causes annual economic losses of up to two billion dollars worldwide^1,2^. MDV is highly oncogenic in its natural host and causes mortalities of up to 100%^1,3^. These high mortality rates are due to clinical disease, virus-induced immunosuppression and T cell lymphomas, which are considered one of the most frequent cancers in animals^4,5^. Moreover, this natural virus-host model is used to investigate virus-induced lymphoma formation. To date, widespread vaccinations with live-attenuated vaccines are the only means to minimize losses caused by MDV^6^. However, vaccines do not provide sterilizing immunity and allow MDV field strains to infect, replicate and shed in vaccinated flocks. In addition, it has been hypothesized that MDV vaccines select for more virulent strains that can overcome the vaccine hurdle^7^.

The MDV genome is a double stranded DNA (dsDNA) of approximately 180 kilo base pairs, containing two unique regions, the unique long (U_L_) and the unique short (U_S_). These two unique regions are flanked by inverted repeats, two internal repeats (long (IR_L_) and short (IR_S_)), and two terminal repeats (long (TR_L_) and short (TR_S_))^8,9^. Like other alphaherpesviruses, MDV also has a complex replication cycle of about 18–20 h^10^ that is thought to be similar to what was observed for other herpesviruses^11,12^. Upon entry into a susceptible cell, the viral genome is released into the nucleus followed by immediate expression of the major viral transactivator ICP4 (infected cell protein 4). ICP4 inhibits the innate cellular defence machinery and serves as a strong transcriptional regulator^13–15^. Next, the DNA polymerase UL30 synthesizes viral genomic DNA as long head to tail concatemers by rolling circle replication^16,17^. Upon packaging of intact viral genomes, tegument proteins including the UL49 are essential for nuclear egress and maturation of assembled viral particles, which acquire their final envelope via budding from trans-Golgi vesicles^11,18,19^.

The CRISPR/Cas9 is an adaptive immune mechanism in bacteria and archaea that targets foreign nucleic acids of invading viruses^20–22^. The CRISPR/Cas9 system consist of the Cas9 nuclease and a guide RNA (gRNA), which targets the complex to complementary DNA sequences that are then cleaved by Cas9^23^. This system has been widely used to edit the genomes of a plethora of cells and was used in many publications on human and animal viruses^24–26^.

Here, we set out to use the CRISPR/Cas9 system to completely abrogate MDV replication. We identified single gRNAs that significantly inhibited virus replication but allowed emergence of escape mutants in some cases. In contrast, simultaneous targeting of two or more MDV genes with gRNAs completely abrogated virus replication and no escape mutants were observed. Thus, our study provides a basis for the application of CRISPR/Cas9 as a powerful tool to block MDV replication.

## Results

### Targeting of essential viral genes impairs MDV replication

**Figure 1:**
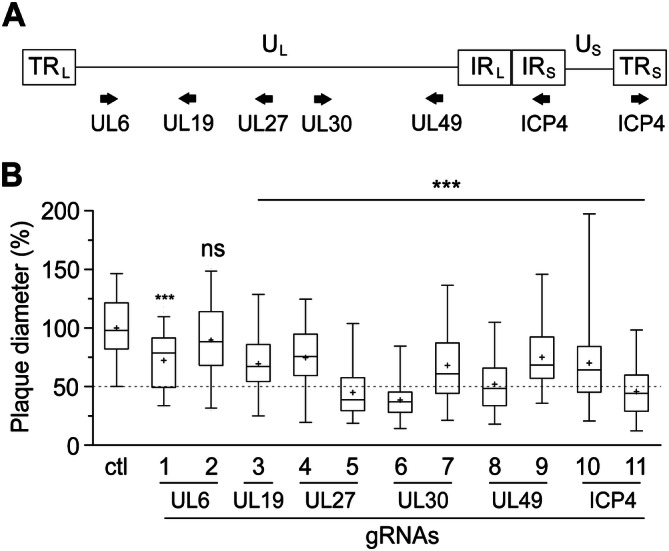
Targeting essential MDV genes by the CRISPR/Cas9 using single gRNAs partially impairs virus replication. (**A**) Overview of the MDV genome showing the target genes. (**B**) Plaque size assays of 11 different gRNAs targeting 6 different MDV essential genes; gRNAs 1 and 2 target the capsid portal protein (UL6, 5′ and 3′); gRNA 3 targets the major capsid protein (UL19, 5′); gRNAs 4 and 5 target the glycoprotein B (UL27, 5′ and 3′); gRNA 6 and 7 target the polymerase protein UL30 (5′ and 3′); gRNAs 8 and 9 target the tegument protein (UL49, 5′ and 3′); and gRNAs 10 and 11 target the infected cell protein (ICP4, 5′ and 3′). Data were analysed by the one-way analysis of variance (ANOVA) with Bonferroni correction and error bars represent the standard deviations (***p ≤ 0.001).

To uncover if the CRISPR/Cas9 system can be used to inhibit replication of the highly cell-associated MDV, we designed gRNAs targeting essential MDV genes. Two independent gRNAs were generated for each of the following genes (Fig. 1A): capsid portal protein (UL6), major capsid protein (UL19), glycoprotein B (UL27), DNA polymerase (UL30), tegument protein UL49, and infected cell protein 4 (ICP4). Alignments of multiple MDV genomes confirmed the high sequence conservation of our selected target sequences. To determine the impact of these gRNAs on MDV replication, we infected cells expressing Cas9 and individual gRNAs with 100 plaque-forming units (pfu) of the very virulent RB-1B strain expressing a GFP reporter. Plaque size assays revealed that individual gRNAs significantly impaired virus replication and spread in the culture (Fig. 1B). Importantly, the gRNAs targeting UL27, UL30, UL49 and ICP4 decreased plaque sizes by more than 50% (p < 0.001, Fig. 1B and S1), highlighting that a single gRNA targeting the MDV genome can strongly impair virus replication.

### Multiple gRNAs completely abrogate virus replication

**Figure 2:**
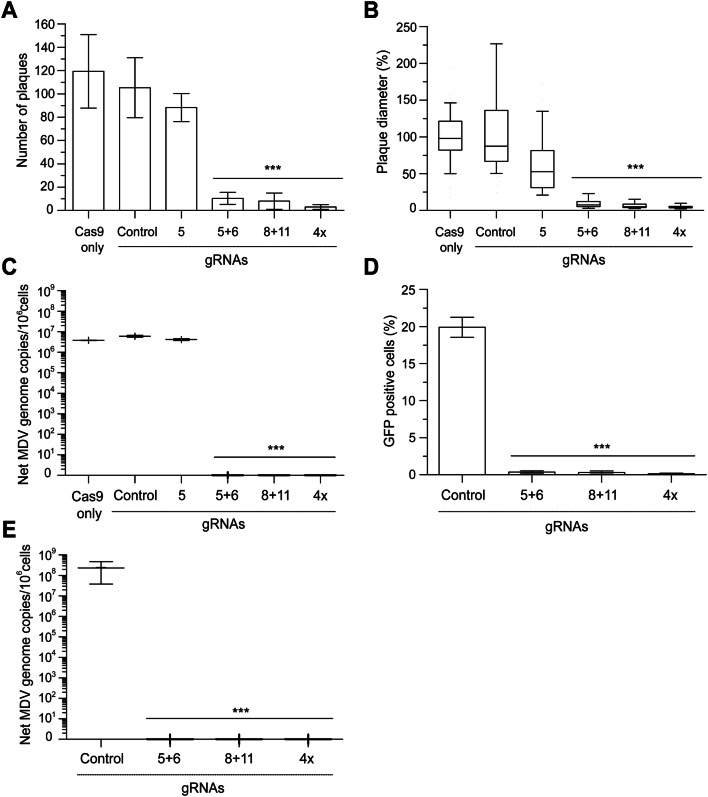
Efficient abrogation of lytic MDV replication. (**A**) Plaque numbers and (**B**) sizes after infection with 100 pfu of the very virulent RB-1B MDV strain. (**C**) Corresponding net increase in MDV genome copies between 0 and 6 days after infection with 100 pfu. (**D**) Percent of MDV-infected cells detected by flow cytometry and (**E**) relative genome copies detected by qPCR at 5 days post-infection with 10,000 pfu. The significant differences between the controls, single gRNAs and the multiplexed gRNAs are indicated with asterisks (***p ≤ 0.001). At least three independent experiments were performed. Data set was analysed by the one-way analysis of variance (ANOVA) with Bonferroni correction and error bars represent the standard deviations.

Next, we tested if combining multiple gRNAs is more effective in blocking MDV replication. To achieve this, we combined the four gRNAs that reduced MDV plaque sizes by more than 50% in pairs (2×) and all together in a single vector (4×). In addition, we used gRNAs targeting the HHV-6 genome as a negative control. To assess the efficiency of multiple gRNAs in inhibiting MDV replication, we infected cells expressing Cas9 and one or more gRNAs with 100 pfu of RB-1B and assessed plaque numbers, plaque sizes and genome replication (Fig. 2). Only very few and small plaques were observed in cells expressing a combination of two or four gRNAs (Fig. 2A). The plaque sizes were significantly decreased by more than 90% (p < 0.001, Fig. 2B) compared to the controls, while single gRNAs only reduced the plaque sizes by 50% as shown in Fig. 1B. In addition, viral genome replication was significantly reduced by about six logs compared to the controls (p < 0.001, Fig. 2C).

To examine if a combination of two or four gRNAs can also protect cells at high infection levels, we infected these cells with 10,000 pfu and measured viral replication by flow cytometry and qPCR. Flow cytometry analysis revealed that two and four combined gRNAs efficiently blocked virus spread in the culture, while replication was not affected in cells expressing a negative control gRNA (Fig. 2D). Moreover, viral genome replication was significantly reduced by about eight logs, underlining that combining two or more gRNAs efficiently blocks MDV replication (p < 0.001, Fig. 2E).

### Multiple gRNAs prevent the emergence of escape mutants

**Figure 3:**
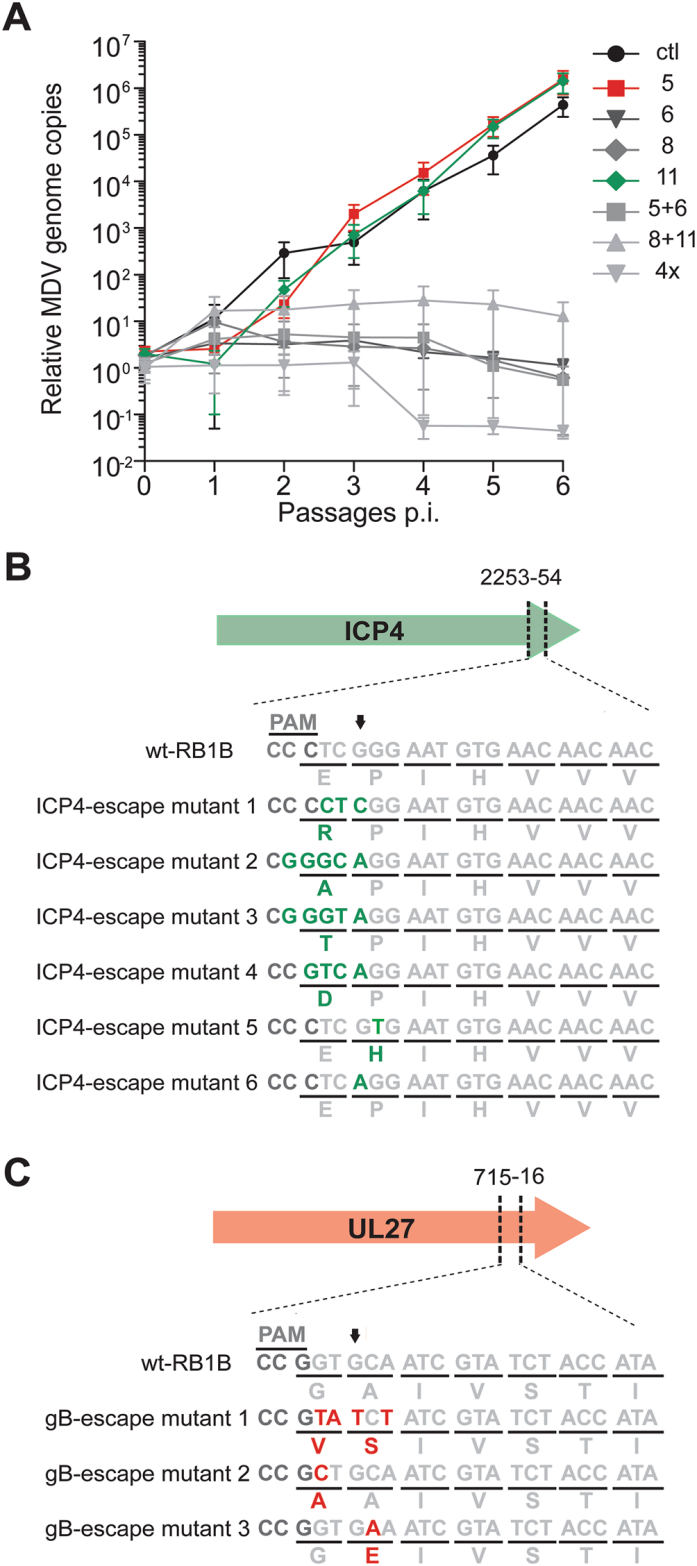
Emergence of MDV escape mutants that evade inefficient single gRNAs. (**A**) qPCR-based multiple-step growth kinetics of MDV in different CRISPR/Cas9 expressing cells upon prolonged infection for up to six passages (33 days). Data are shown as average of three independent experiments and error bars represent the standard deviations (p ≤ 0.001, ctl vs. 6, 8, 5 + 6, 8 + 11 and 4×; Kruskal–Wallis test). (**B**) Analysis of sequences of the MDV variants detected in the single gRNA11 and (**C**) gRNA 5, both targeting the 3′ ends of ICP4 and UL27, respectively. The sequences on the top correspond to wild-type (wt) RB-1B sequences and at the bottom to sequences of detected CRISPR/Cas9 escape mutants. Numbers above the arrows indicate the positions of the amino acid substitutions in the respective open reading frame. Arrows at + 3 positions after the protospacer adjacent motif (PAM) site refer to the Cas9 cleavage site.

To determine if escape mutants arise upon serial passaging, we infected CRISPR/Cas9 expressing cells with 10,000 pfu, cultured them for six passages and assessed virus replication by qPCR. Escape mutants evolved in cultures containing single gRNAs targeting the 3-prime (3′) end of UL27 and ICP4, resulting in viruses that replicated comparable to wild-type virus (Fig. 3A). Interestingly, we did not observe escape mutants for single gRNAs targeting the 5′ end of UL30 and UL49. More importantly, no escape mutants arose upon combination of two or four gRNAs. To ensure that indeed no escape mutants can arise using multiple gRNAs, we infected cells with a high dose, splitted them only 1:2 and maintained them for 10 passages. No plaques arose in cells expressing the 2 × and 4 × gRNA cassettes, while escape mutants arose with single gRNAs against the 3′ end of UL27 and ICP4 (Fig. 3B,C). Notably, we excluded the presence of these mutations in the RB-1B wild-type virus stock used in this study by Sanger sequencing.

To examine how the virus could escape from the Cas9 cleavage, we assessed the cleavage site of the escape mutants by Sanger sequencing. Strikingly, we always found mutations in the gRNA target sequence of both UL27 and ICP4 (Fig. 3B,C). These mutations resulted in either one or two amino acid substitutions and still allowed efficient virus replication (Fig. 3A). Taken together, our data demonstrate that the use of single gRNAs can result in the escape of recombinant viruses and that a combination of two or more gRNAs can completely abrogate replication of this highly cell-associated herpesvirus.

## Discussion

In this study, we provide the first proof-of-concept that the CRISPR/Cas9 system can abrogate replication of a highly cell-associated herpesvirus. We established different CRISPR/Cas9 cell lines expressing single or multiplexed gRNAs that target six essential MDV genes (Fig. 1A). Intriguingly, virus replication and spread were impaired by up to 50% using single gRNAs (Fig. 1B) and were completely abrogated using multiple gRNAs (Fig. 2). It is well known that not all gRNAs work equally well or at all, however the reason for that is not fully understood^27,28^. Therefore, it was not surprising that some gRNAs performed better than others.

It has been previously shown that the CRISPR/Cas9 can interfere with the herpesvirus life cycle in different ways^26^. First, it can impair the packaging of intact viral genomes through induction of DNA double-strand breaks (DSBs) in the viral genome. Moreover, it can disrupt the expression of the targeted essential proteins. Finally, it may indirectly affect viral replication as the introduced DSBs are repaired by the non-homologous end joining (NHEJ) DNA repair, which often produces deleterious mutations that can hamper subsequent rounds of viral infections^26^. Nevertheless, the additive effect of combining multiple gRNAs is more stringent because it could result in the loss of large parts of viral genome^26^, shifting the balance between DSB repair and Cas9 DNA cleavage. This likely leads to a subsequent loss of virus genome integrity and fragments that cannot replicate^12^. In addition, abrogation of the expression of multiple essential genes blocks the formation of infectious viral particles^26,29^. Together, our results are in agreement with a previous report that demonstrated the inhibition of herpesviruses replication by simultaneous CRISPR/Cas9 targeting of multiple genes^26^.

Until now, only limited data is available on the prevention of viral escape mutants using multiple gRNA targets. A few studies reported the efficiency of the multiplexed gRNAs to prevent or delay the evolution of HIV, which has very high evolutionary rates in contrast to dsDNA viruses^29,30^. In this report, we assessed if escape mutants can emerge upon CRISPR/Cas9 targeting of multiple MDV genes and could not detect any viral breaks in more than 30 days of culture. This is likely due to the high efficiency of the multiplexed gRNAs against MDV and low evolutionary rates.

Interestingly, we observed that MDV could restore wild-type replication levels in case of two single gRNAs representing escape mutants that targeted UL27 and ICP4, respectively (Fig. 3). It is tempting to speculate that MDV may exploit the error-prone NHEJ DNA repair mechanism to mutate the targeting site while maintaining the function of the respective essential viral gene. These escape mutants rapidly accumulated over time and lead to a restoration of the wild-type replication phenotype. In fact, the CRISPR/Cas9 recognition step is a very specific process where non-specific binding is usually transient and short-lived. Therefore, these nucleotide substitutions at the protospacers (Fig. 3B,C) are expected to interfere with one of the crucial steps of this system, the recognition phase^31^. We selected essential genes to minimize the emergence of escape mutants, as only mutations that result in a functional protein are tolerated by the virus. Targeting non-essential genes or sequences would likely allow a rapid repair and escape from the negative selection^32^. Overall, our findings agree with previous reports that showed accelerated viral escape upon inefficient targeting by single gRNAs^26,29,33^.

In summary, our study not only emphasizes the potency of the CRISPR/Cas9 system against one of the deadliest oncogenic herpesviruses but also paves the way for future in vivo applications.

## Materials and methods

### Cells and viruses

Primary chicken embryo cells (CEC) cells were isolated from 11-day-old specific-pathogen-free (SPF) chicken embryos (VALO Biomedia, Germany) as previously described^34,35^. The cells were maintained in Eagle’s minimal essential media (MEM; PAN Biotech, Germany) with 1–10% foetal bovine serum (FBS; PAN-Biotech) and 100 U/ml penicillin and 100 μg/ml streptomycin (AppliChem, Germany) at 37 °C and 5% CO_2_.

The duck embryo retina-derived cell line CR and 293 T human embryonic kidney cells were maintained in Dulbecco’s MEM/Ham’s F-12 (1:1, Biochrom, Germany) and RPMI 1,640 (PAN-Biotech, Germany) respectively^36^. All media were supplemented with glutamine and NaHCO_3_ (PAN-Biotech, Germany, 10% heat-inactivated FBS (PAN-Biotech) and 100 U/ml penicillin and 100 μg/ml streptomycin (AppliChem) and cells were maintained at 37 °C and 5% CO_2_. We used the very virulent RB-1B GFP reporter strain for all infections^37,38^. The virus was reconstituted by calcium phosphate transfection of CEC with purified bacterial artificial chromosome DNA as previously described^39^ and propagated on fresh CEC. Virus stocks (passage 7) were frozen in liquid nitrogen and titrated before use.

### CRISPR/Cas9 DNA vectors

To deliver the CRISPR/Cas9 system to the cells, we used two different lentiviral CRISPR/Cas9 transfer vectors. The pSicoR-CRISPR-PuroR vector was used to deliver the wild-type *S. pyogenes* Cas9 protein to the cells. This vector has a Cas9 gene, N-terminally fused to PuroR via a T2A-ribosome skipping sequence and expressed under the control of the human EF1A promoter^26,40^. The pLKO5.sgRNA.EFS.PAC vector (Addgene: #57,825) was used to deliver the different MDV-specific gRNAs to the cells^41^. Of note, this vector was modified by exchanging the PuroR cassette for HygroR using the flanking BamHI and MluI sites.

### Generation of anti-MDV CRISPR/Cas9-expressing cell lines

The online algorithm tool https://chopchop.cbu.uib.no/^42,43^ was used to identify the CRISPR RNAs with highest specificity for MDV and no off-targets in chickens. Two independent gRNAs were designed for each selected essential MDV gene (Table 1). The RNAs were cloned into the pLKO5.sgRNA.EFS.PAC vector using a primer set harbouring BsmB-I restriction sites (Table S1). For the construction of multiplexed gRNA vectors (5 + 6), (8 + 11), and (4×), the Q5 high-fidelity DNA polymerase and restriction sites SalI and XhoI (New England Biolabs, MA, USA) were used to clone the single and dual gRNA cassettes into the pLKO5.sgRNA.EFS.PAC vector (Table S1). Sequence analyses were carried out using the Vector NTI Advance 9.1 software package (Life Technologies, CA, USA). The positive clones were stored as glycerol stocks in − 80 °C until further use.

**Table 1:** gRNA target sequences in the MDV genome used in this study.

**Construct**	**gRNA target sequence in MDV**	**MDV target gene**
1	TTAGGATATACTGATGGCCA	Capsid portal protein (UL6)
2	TAATTCGGGAAGGCAACGCG	
3	CACTTCAGATAATAATGCGA	Major capsid protein (UL19)
4	GGTTCGGGACATTTTCGCGG	Glycoprotein B (UL27)
5	TATGGTAGATACGATTGCAC	
6	AATGGCTTATCATTTCCAC	DNA polymerase (UL30)
7	ATGTTCACAACGATACGAAG	
8	GACGTTTCGTCTACCACCCG	Tegument protein (UL49)
9	TCTGAACGTACAAGACGCGG	
10	GAGGCAATTGGCAGATACGG	Infected cell protein (ICP4)
11	GTTGTTGTTCACATTCCCGA	
gRNA control	GGAGTAGTGTTTGACGGCCA	HHV6 tegument protein (UL25)

The pSicoR-CRISPR-PuroR lentiviral transfer vector and two third-generation lentiviral packaging plasmids were used to transfect the 293 T cells following the standard lentiviral production protocol^44^. These lentiviruses were used to transduce the CR cells via spin infection at 1,200 × *g* for 2 h at room temperature. The Cas9-transduced cells were subjected to puromycin selection (1 µg/ml; Carl Roth, Germany) for 3–4 days and Cas9-expression was confirmed by fluorescent microscopy and FACS using the mouse monoclonal α-D tag antibody (ABM, Canada) and the secondary goat anti-mouse IgG Alexa Fluor 488 antibody (Invitrogen, CA, USA; Fig. S2). Lipofectamine 2000 (Invitrogen, MA, USA) was used to transfect the Cas9-transduced cells with different single or multiplexed gRNAs vectors following the manufacturer’s instructions. The gRNA-transfected Cas9 cells were selected with hygromycin (200 µg/ml; Carl Roth, Germany) for 6 days. CRISPR/Cas9 CR cells were then expanded and frozen in liquid nitrogen until further use.

### Plaque size assays

To test the efficacy of the CRISPR/Cas9 system against spread and replication of MDV, different CRISPR/Cas9 cell lines were infected with 100 pfu of the RB-1B GFP reporter virus. Six days post-infection (dpi), plaque numbers were counted and 50 randomly selected plaques per well were imaged and measured using the ImageJ software (NIH; https://imagej.nih.gov/ij/) as previously described^45,46^. Plaque diameters were calculated and compared to the respective control. At least three independent experiments were performed.

### Quantitative PCR (qPCR)

To assess MDV genome copies by qPCR, DNA of infected cells was extracted with the RTP DNA/RNA Virus Mini Kit (Stratec Molecular, Germany). MDV copy numbers were determined as previously described^47^. Briefly, MDV genome copies were measured using a set of specific primers and a probe that target ICP4. The inducible nitric oxide synthase (iNOS) was used to normalize the MDV ICP4 copy numbers as previously published^47^.

### Flow cytometry

Cas9/gRNA cell lines were infected with 10,000 pfu of RB-1B expressing GFP. At 5 dpi, the cells were analysed by flow cytometry (CytoFlex S; Beckman Coulter, CA, USA) to detect the percentage of GFP positive cells. At least 10,000 living cells were measured for each independent experiment and analysed using the CytExpert software (version 2.3; Beckman Coulter).

### Multi-step growth kinetics assays

CRISPR/Cas9 cell lines expressing one or multiple gRNAs were infected with 10,000 pfu of RB-1B and passaged at a ratio of 1:15 for six passages. Genome replication was assessed at each passage by qPCR as previously published^47^. Growth kinetics were determined in three independent experiments.

### CRISPR/Cas9 escape mutants

CRISPR/Cas9 cell lines expressing one or multiple gRNAs were infected with 10,000 pfu and passaged at a ratio of 1:2 every three days up to 33 dpi. Afterwards, the cells were subjected to a total viral DNA extraction and the genomic targets of the respective gRNAs were amplified and analysed by Sanger sequencing to screen for potential CRISPR/Cas9-escape mutants.

### Ethics statement

The in vitro work was approved by the *Landesamt für Gesundheit und Soziales* Berlin, Germany (S2-Genanlage 919/94) and all methods were carried out in accordance with relevant guidelines and regulations.

### Statistical analysis

The data were analysed with GraphPad Prism (version 8; GraphPad Software, Inc., CA, USA). The data were assessed for normal distribution and subsequently analysed using the one-way analysis of variance (ANOVA) with Bonferroni correction. P values ≤ 0.05 were considered significant.

## Supplementary information

Supplementary file1 (DOCX 1006 kb)
