# Supplementary material for: Abrogation of Marek’s disease virus replication using CRISPR/Cas9
Source: Sci Rep. 2020 Jul 2;10:10919. doi: 10.1038/s41598-020-67951-1 (PMC7331644; doi:10.1038/s41598-020-67951-1)
Supplement: Supplementary file 1 — Supplementary file1 (DOCX 1006 kb) [file 41598_2020_67951_MOESM1_ESM.docx]

**Abrogation of Marek’s disease virus replication using CRISPR/Cas9**

**Supplementary information**

Ibrahim T. Hagag^1,2^, Darren J. Wight^1^, Denise Bartsch^3^, Hicham Sid^3^, Ingo Jordan^4^, Luca D. Bertzbach^1^, Benjamin Schusser^3^ and Benedikt B. Kaufer^1^

^1^ Institut für Virologie, Freie Universität Berlin, Robert-von-Ostertag-Str. 7-13, 14163 Berlin, Germany

^2^ Department of Virology, Faculty of Veterinary Medicine, Zagazig University, El-Tagneed St. 114, 44511 Zagazig, Egypt.

^3^ Reproductive Biotechnology, School of Life Sciences Weihenstephan, Technical University of Munich, Liesel-Beckmann-Str. 1, 85354 Freising, Germany.

^4^ ProBioGen AG, Herbert-Bayer-Straße 8, 13086 Berlin, Germany.

**Tab. S1:** Primers used to clone gRNAs and multiplex vectors

| **Gene/s** | **Construct/s** | **Direction** | **Sequences (5`🡪3`)** |
| --- | --- | --- | --- |
| UL6 | 1 | For | CACCGTTAGGATATACTGATGGCCA |
|  |  | Rev | AAACTGGCCATCAGTATATCCTAAC |
|  | 2 | For | CACCGTAATTCGGGAAGGCAACGCG |
|  |  | Rev | AAACCGCGTTGCCTTCCCGAATTAC |
| UL19 | 3 | For | CACCGCACTTCAGATAATAATGCGA |
|  |  | Rev | AAACTCGCATTATTATCTGAAGTGC |
| UL27 | 4 | For | CACCGGGTTCGGGACATTTTCGCGG |
|  |  | Rev | AAACCCGCGAAAATGTCCCGAACCC |
|  | 5 | For | CACCGTATGGTAGATACGATTGCAC |
|  |  | Rev | AAACGTGCAATCGTATCTACCATAC |
| UL30 | 6 | For | CACCGAATGGCTTATCATTTTCCAC |
|  |  | Rev | AAACGTGGAAAATGATAAGCCATTC |
|  | 7 | For | CACCGATGTTCACAACGATACGAAG |
|  |  | Rev | AAACCTTCGTATCGTTGTGAACATC |
| UL49 | 8 | For | CACCGGACGTTTCGTCTACCACCCG |
|  |  | Rev | AAACCGGGTGGTAGACGAAACGTCC |
|  | 9 | For | CACCGTCTGAACGTACAAGACGCGG |
|  |  | Rev | AAACCCGCGTCTTGTACGTTCAGAC |
| ICP4 | 10 | For | CACCGGAGGCAATTGGCAGATACGG |
|  |  | Rev | AAACCCGTATCTGCCAATTGCCTCC |
|  | 11 | For | CACCGGTTGTTGTTCACATTCCCGA |
|  |  | Rev | AAACTCGGGAATGTGAACAACAACC |
| UL27, UL30, UL49 +ICP4 | (5+6) (8+11) 4x | Rev | CCCGTTGCGAAAAAGAACG |
|  | (5+6) (8+11) | For | CTCCTGTCGACTTTCCCATGATTCCTTCATATTTG |
|  | 4x | For | CTCCTGTCGACGGCAAGTTTGTGGAATTGGTTTAAC |


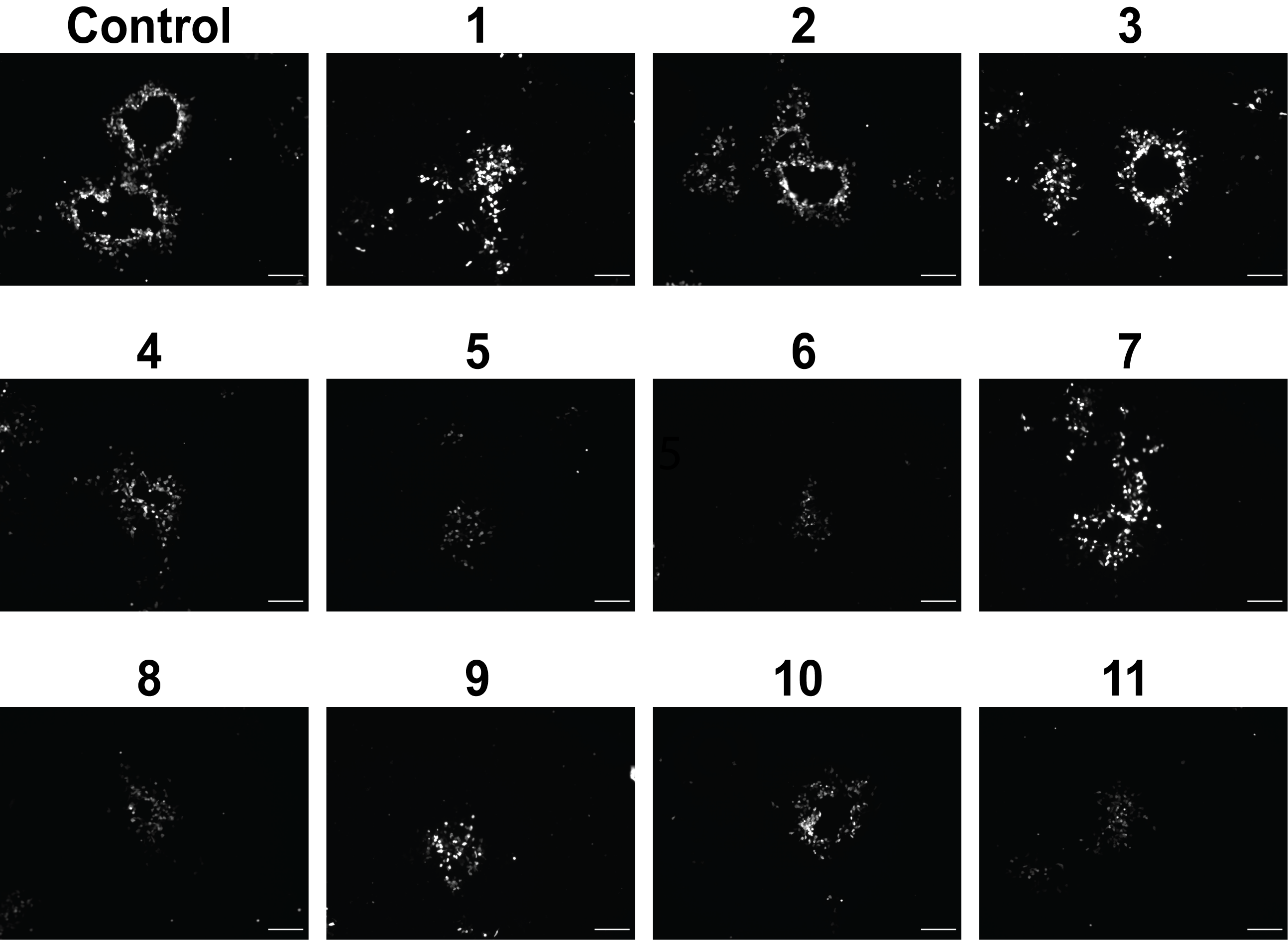


**Fig. S1:** Representative plaque images. Plaque images from RB-1B-infected CR cells without (control) and with indicated gRNAs (1-11). The scale bars correspond to 1000µM.


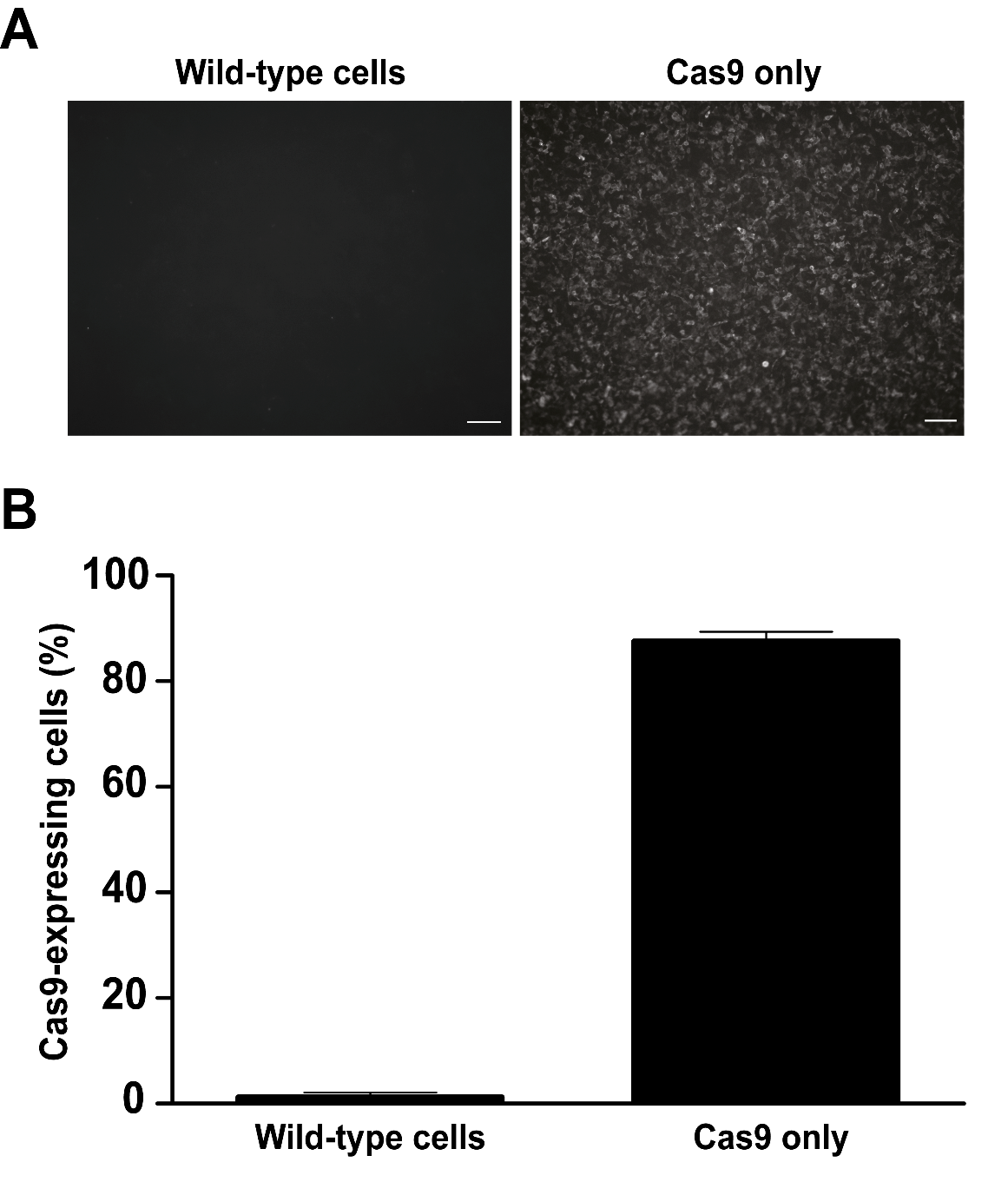


**Fig. S2:** Cas9 expression in CR cells. **(A)** Immunofluorescence assessment of control CR cells and Cas9-expressing polyclonal CR cells. The scale bars correspond to 100µM **(B)** FACS analysis of Cas9 expression in control CR cells and Cas9-expressing representative monoclonal CR cells (n=3). Error bars represent standard deviations.
